# A 1-Cys Peroxiredoxin from a Thermophilic Archaeon Moonlights as a Molecular Chaperone to Protect Protein and DNA against Stress-Induced Damage

**DOI:** 10.1371/journal.pone.0125325

**Published:** 2015-05-01

**Authors:** Sangmin Lee, Baolei Jia, Jinliang Liu, Bang Phuong Pham, Jae Myeong Kwak, Yuan Hu Xuan, Gang-Won Cheong

**Affiliations:** 1 Division of Applied Life Sciences and Research Institute of Natural Science, Gyeongsang National University, Jinju, Korea; 2 Division of Electron Microscopic Research, Korea Basic Science Institute, Daejeon, Korea; 3 Department of Life Sciences, Chung-Ang University, Seoul, Korea; 4 College of Plant Sciences, Jilin University, Changchun, China; 5 College of Pharmaceutical Sciences, Wenzhou Medical University, Wenzhou, China; Instituto de Biociencias - Universidade de São Paulo, BRAZIL

## Abstract

Peroxiredoxins (Prxs) act against hydrogen peroxide (H_2_O_2_), organic peroxides, and peroxynitrite. *Thermococcus kodakaraensis* KOD1, an anaerobic archaeon, contains many antioxidant proteins, including three Prxs (Tk0537, Tk0815, and Tk1055). Only Tk0537 has been found to be induced in response to heat, osmotic, and oxidative stress. Tk0537 was found to belong to a 1-Cys Prx6 subfamily based on sequence analysis and was named 1-Cys TkPrx. Using gel filtration chromatography, electron microscopy, and blue-native polyacrylamide gel electrophoresis, we observed that 1-Cys TkPrx exhibits oligomeric forms with reduced peroxide reductase activity as well as decameric and dodecameric forms that can act as molecular chaperones by protecting both proteins and DNA from oxidative stress. Mutational analysis showed that a cysteine residue at the N-terminus (Cys^46^) was responsible for the peroxide reductase activity, and cysteine residues at the C-terminus (Cys^205^ and Cys^211^) were important for oligomerization. Based on our results, we propose that interconversion between different oligomers is important for regulating the different functions of 1-Cys TkPrx.

## Introduction

*Thermococcus kodakaraensis* KOD1, a model thermophilic organism whose entire genome is sequenced, is a thermophilic anaerobic archaeon belonging to the *Thermococcaceae* family [1]. As an anaerobe living in deep-vent environments, *Thermococcus* encounters high levels of oxygen stress in the water surrounding the vent [2, 3]. In anaerobes and microaerophiles, peroxiredoxins (Prxs) play an important role in protecting organisms from oxidative and nitrosative stress through their peroxidase and peroxynitrite reductase activities, using thioredoxin, cyclophilin, and glutaredoxin as reducing equivalents [4, 5]. In addition, Prxs can also promote H_2_O_2_-mediated cell signalling [6], and some can also act as efficient molecular chaperones [7].

Prxs can be classified by the number of cysteine residues in their catalytic cycle and by their enzymatic mechanisms, which together give rise to the “1-Cys”, “atypical” and “typical 2-Cys” categories [8]. All Prxs share a common basic catalytic mechanism, where conserved cysteine residues (Cp) in the N-terminus are oxidized to sulfenic acid (Cys-SpOH) by a peroxide substrate (9, 10). In the typical 2-Cys Prxs, the peroxidatic (C_P_) and resolving (C_R_) cysteines are located on different subunits, and the C_P_ attacks an O-O bond of the peroxide (ROOH) substrate to form the product ROH and the sulfenic derivative C_P_-SOH. This sulfenic derivative then forms a disulfide bond (C_P_-S-S-C_R_) with the other conserved cysteine residues (C_R_) [5, 9]. In the atypical 2-Cys Prxs, two conserved cysteine residues (C_P_ and C_R_) are located on the same subunit of the polypeptide chain. The catalytic cycle passes through an intermediate inter- [10] or intramolecular [11] disulfide bond that can be reduced by a dithiol oxidoreductase, such as thioredoxin. In contrast to the 2-Cys Prxs, 1-Cys Prxs do not contain a resolving cysteine, and thus reduction of the sulfenic acid intermediate involves another external electron donor, such as thioredoxin, ascorbic acid [12], the GRX-GSH system [13], or the GST-GSH system [14]. Recently, an alternative mechanism involving three cysteines has been proposed for the AhpC Prxs from *Mycobacterium tuberculosis* [15] and a Prx from *Aeropyrum pernix* K1 [16].

In this study, we found that TK0537 from *Thermococcus kodakarensis* KOD1 is a 1-Cys Prx (1-Cys TkPrx) containing three cysteine residues and demonstrated that 1-Cys TkPrx displays different oligomeric structures under different redox states. Furthermore, various activities (peroxidase, molecular chaperone, and DNA binding) of the 1-Cys TkPrx were measured in different redox states. The results showed that oxidized or overoxidized 1-Cys TkPrx can prevent protein aggregation and DNA damage from oxidative and thermal stress. Finally, the relationship between the structure and function of 1-Cys TkPrx is discussed.

## Materials and Methods

### Cell strains

*T*. *kodakaraensis* KOD1, which was kindly donated by the Japan Collection of Microorganisms, RIKEN BioResource Center, Japan, was used to prepare crude cell extracts and to isolate genomic DNA [10, 11]. Heat, oxidative, and salt stressors were applied according to previously published methods [17].

### Two-dimensional electrophoresis and MALDI-TOF MS

Two-dimensional electrophoresis (2DE) was performed under various stress conditions such as osmotic, heat, and oxidative stress. After electrophoresis, proteins were detected by silver staining. Stained gels were scanned and digitized using a Duoscan (Agfa, NJ, USA) scanner. Protein spots were evaluated using PDQuest version 7.1 software (Bio-Rad, CA, USA), and notably overexpressed proteins were excised using a sterile pipette tip and processed further for MALDI-TOF-MS analysis. We performed searches in the NCBI, SwissProt/TrEMBL, and MSDB sequence databases using MS-Fit Mascot and ExPASy to identify proteins [17].

### Expression and Purification of 1-Cys TkPrx

The *Tk0537* gene was amplified from *T*. *kodakaraensis* KOD1 genomic DNA by PCR and cloned into the pET28(a) expression vector. The primers (Forward primer: 5’-CAT ATG GTC GTC ATA GGA AAG T-3’, Reverse primer: 5’-AAG CTT TCA CTC GAG CTT CTT GTA GCA-3’) were designed based on the *T*. *kodakaraensis* KOD1 genome sequence. The PCR product and pET28(a) plasmid were digested by *Nde* I and *Hind* III, and the digested products were then ligated by DNA ligase. The ligation products were transformed into *Escherichia coli* BL21(DE3) pLys by electroporation. Finally, the recombinant plasmid was confirmed by DNA sequencing. Recombinant *E*. *coli* cells (2 L) were cultured in Luria-Bertani (LB) medium containing 50 μg·mL^-1^ kanamycin (Sigma-Aldrich) to the logarithmic phase (OD_600_ = 0.6) and then induced for 4 h with 1 mM isopropyl-β-D-thiogalactopyranoside (IPTG) at 37°C. The cells were harvested and resuspended with lysis buffer (50 mM Tris-HCl, 0.1 M KCl, 5 mM imidazole, and 10% glycerol, pH 8.0). After disrupting the cells by sonication, the samples were heated at 65°C for 30 min. The heat-stable supernatants were separated by centrifugation (13000 rpm, 20 min) and loaded onto a Profinity IMAC Ni-charged resin (Bio-Rad, CA, USA), which was pre-equilibrated with lysis buffer. Recombinant 1-Cys TkPrx was eluted with approximately 100 mM imidazole. Standard procedures were used to examine the purity of the proteins by SDS-PAGE.

### Gel filtration chromatography

The gel filtration chromatography assays were performed using a DuoFlowTM chromatography system with a HiLoad 10/60 Superdex 200 column (GE Healthcare, WI, USA). The column was equilibrated with 50 mM Tris-HCl buffer (pH 7.4) containing 300 mM NaCl, and 0.5 mM EDTA. Protein samples (5 mg) were loaded onto the column for fractionation. Blue dextran (2000 kDa), thyroglobulin (699 kDa), ferritin (440 kDa), catalase (232 kDa), and ovalbumin (43 kDa) (GE Healthcare, WI, USA) were used as the standard proteins.

### Blue-Native (BN) PAGE analysis

Analysis with BN gels was carried out as previously described [18] and performed on a Protean II minigel system (Bio-Rad, CA, USA). Anode and cathode buffers contained 50 mM Tris and 75 mM glycine, and only the cathode buffer was supplemented with 0.002% Serva blue G. Before loading the sample, 2 μl of sample buffer (500 mM 6-amino-n-capric acid, 5% Serva blue G) was added. The gel was run overnight at 4°C. Thyroglobulin (669 kDa), ferritin (440 kDa), catalase (232 kDa), alodolase (158 kDa), and albumin (66 kDa) were used as molecular weight size standards (GE Healthcare, WI, USA).

### Mutagenesis

The primers used to generate the single cysteine-to-serine mutations were: C46S, forward primer, 5’- CCG GCT GAC TTC ACC CCG GTC AGC ACG ACC GAG TTC TAC GCC ATG -3’, C46S, reverse primer, 5’- CAT GGC GTA GAA CTC GGT CGT GCT GAC CGG GGT GAA GTC AGC CGG-3’; C205S, forward primer, 5’- AAG GCC AAG GGC GAG ATC GAG AGC TAC GAC TGG TGG TTC TGC TAC -3’, C205S, reverse primer, 5’- GTA GCA GAA CCA CCA GTC GTA GCT CTC GAT CTC GCC CTT GGC CTT -3’; and C211S, forward primer, 5’- GAG TGC TAC GAC TGG TGG TTC AGC TAC AAG AAG CTC GAG TGA -3’, C211S, reverse primer: 5’—TCA CTC GAG CTT CTT GTA GCT GAA CCA CCA GTC GTA GCA CTC -3’. The PCR was performed using *Pfu* polymerase (Takara, Kyoto, Japan), and the cycling parameters were as follows: 95°C for 5 min (1 cycle), 95°C for 30 sec, and 68°C for 12 min (12 cycles). After amplification, the PCR mixture was digested by *DpnI* and transformed into an *E*. *coli* BL21 (DE3) pLys strain. The mutations were confirmed by DNA sequencing.

### Aggregation assays

For light scattering experiments, 1-Cys TkPrx was freshly prepared to avoid freeze-thaw and cryopreservation artefacts. The aggregation of *Thermus flavonus* malate dehydrogenase (MDH) was monitored by measuring the apparent absorption due to light scattering in a spectrophotometer. MDH was incubated in 50 mM Hepes (pH 8.0) buffer at 45°C or 95°C with various concentrations of 1-Cys TkPrx. Thermal aggregation of the substrate was determined by monitoring the increase in turbidity at an absorbance of 340 nm [19].

### DNA protection assays

DNA protection assays of 1-Cys TkPrx were performed using the metal-catalysed oxidation (MCO) system as described by Lim et al [20] at 95°C. In the MCO system, the reaction mixtures (50 μL), consisting of 3 μM FeCl_3_, 10 mM DTT and different concentrations of 1-Cys TkPrx, were incubated for 2 h at 37°C before 200 ng of pET28(a) plasmid DNA was added. The reaction was initiated by incubating the DNA mixture at the same temperature for 2 h. In the high temperature condition, 200 ng of pET28(a) plasmid DNA and 600 ng of 1450 bp double-stranded DNA (dsDNA) were mixed with different concentrations of 1-Cys TkPrx. The mixtures were incubated at 95°C for 2 h. The ability of 1-Cys TkPrx to protect DNA was evaluated on a 1% (w/v) agarose gel after staining with ethidium bromide.

### Peroxidase Activity assays

Peroxidase activity was measured using the ferrous oxidation xylenol (FOX) assay [21, 22]. Incubation mixtures (100 μl total volume) containing the following: 50 mM HEPES buffer (pH 7.4), various concentrations of 1-Cys TkPrx, and 1 mM H_2_O_2_, or tert-butyl hydroperoxide (t-BOOH) were incubated at 85°C for 10 min in the presence or absence of 1 mM DTT. The reaction was stopped by the addition of ice-cold FOX reagent, and the absorbance was measured at 560 nm. The amount of H_2_O_2_ was calculated from the sample absorption using a molar extinction coefficient (ε) of 2.24 × 10^5^ M^-1^cm^-1^. The parameters (with standard deviation) were determined for three separate experiments.

### Electron microscopy and image processing

Five microliters of the proteins, wild type, C46S, C205S, C211S, and DTT- or H_2_O_2_-treated wild type proteins, was applied to freshly glow-discharged carbon-coated copper grids, and the grids were immediately negatively stained using 1% uranyl acetate. The grids were examined using a FEI Tecnai transmission electron microscope (TEM) operated at 120 kV, and the images were recorded on Kodak film at a nominal magnification of 42000x. For single-particle analysis, the images of individual particles were interactively selected in the micrographs, windowed out, and imported into the SEMPER [23] and EM [24] software packages. A total of 1638 wild type, 780 H_2_O_2_-treated, 951 DTT-treated, 1205 C46S mutant, 122 C205S mutant, and 176 C211S mutant particles were used for image processing. The selected particles were translationally and rotationally aligned using standard correlation methods [25]. To analyse the rotational symmetry of images viewed from the top, the aligned images were subjected to multivariate statistical analysis. The individual images were translationally and rotationally aligned using standard correlation methods [26].

## Results

### 1-Cys TkPrx is up-regulated in response to different stressors

**Fig 1:**
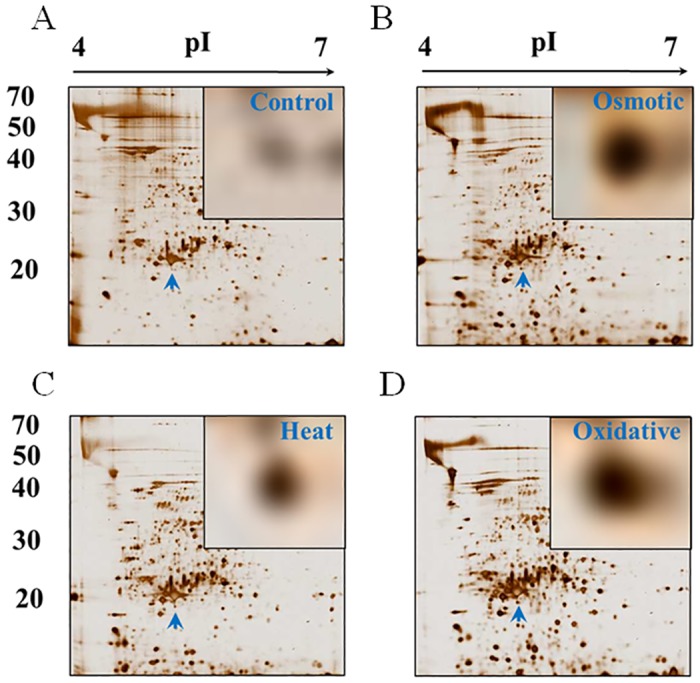
Differential Expression of Proteins in *T*. *kodakarensis* KOD1 Induced by Various Stressors. Comparison of the expression patterns of total protein. A, non-treatment (control); B, osmotic shock (1 M NaCl); C, heat shock (95°C for 1 h); D, oxidative shock (addition of oxygen (5 L/min) for 30 min). Arrows (sky blue) and enlarged views in each figure indicate the expression level of 1-Cys TkPrx under the different stress conditions.

In its natural environment, *Thermococcus* faces various types of stress, including oxidative, heat, cold, and osmotic stress. Previously, we used 2-DE to identify stress-induced proteins in *T*. *kodakaraensis* KOD1 in response to heat (95°C), osmotic (1 M NaCl) and oxidative (O_2_) challenges (Fig 1) [17]. MALDI-TOF MS analysis identified 59, 42 and 29 proteins that were up-regulated in response to heat, osmotic and oxidative stress, respectively (data not shown). Among these, a 1-Cys Prx (Tk0537, named 1-Cys TkPrx) was overexpressed in all three conditions (Fig 1). Because only 1-Cys TkPrx was up-regulated by the various stressors among the three Prx proteins (Tk0537, Tk0815, and Tk1055) in *T*. *kodakaraensis* KOD1, we cloned *1-Cys TkPrx* (*Tk0537*) and overexpressed it in *E*. *coli* BL21(DE3) pLys for further investigation.

### Cloning and sequencing of 1-Cys TkPrx

The DNA sequence encoding 1-Cys TkPrx (Tk0537) has an open reading frame of 651 nucleotides, indicating that it is composed of 216 amino acids, with a predicted molecular mass of 24.6 kDa. Analysis by PREX, which is a database currently comprised of 3516 Prxs, showed that 1-Cys TkPrx belongs to the Prx6 subfamily. 1-Cys TkPrx homologues were found using BLAST in the NCBI database, and the sequences were aligned using Clustal W. The results showed that 1-Cys TkPrx shares a significant degree of identity with 1-Cys Prxs from human (38%), mouse (37%), *Arabidopsis* (38%), and yeast (36%) (S1 Fig). The N-terminal cysteine of 1-Cys TkPrx is located at a position similar to the position of the cysteine residues in these proteins. Interestingly, 1-Cys TkPrx contains two additional cysteines in its C-terminus. 1-Cys Prx proteins with three cysteine residues have also been found in other thermophilic organisms, such as *Thermococcus barophilus*, *Aeropyrum pernix* K1, *Pyrococcus horikoshii*, and *Methanocaldococcus vulcanius*. The two C-terminal cysteines appear to be highly conserved as a CXDWWFC motif in these organisms, though the function of these cysteine residues has not yet been determined.

### Overview of the structure of 1-Cys TkPrx

**Fig 2:**
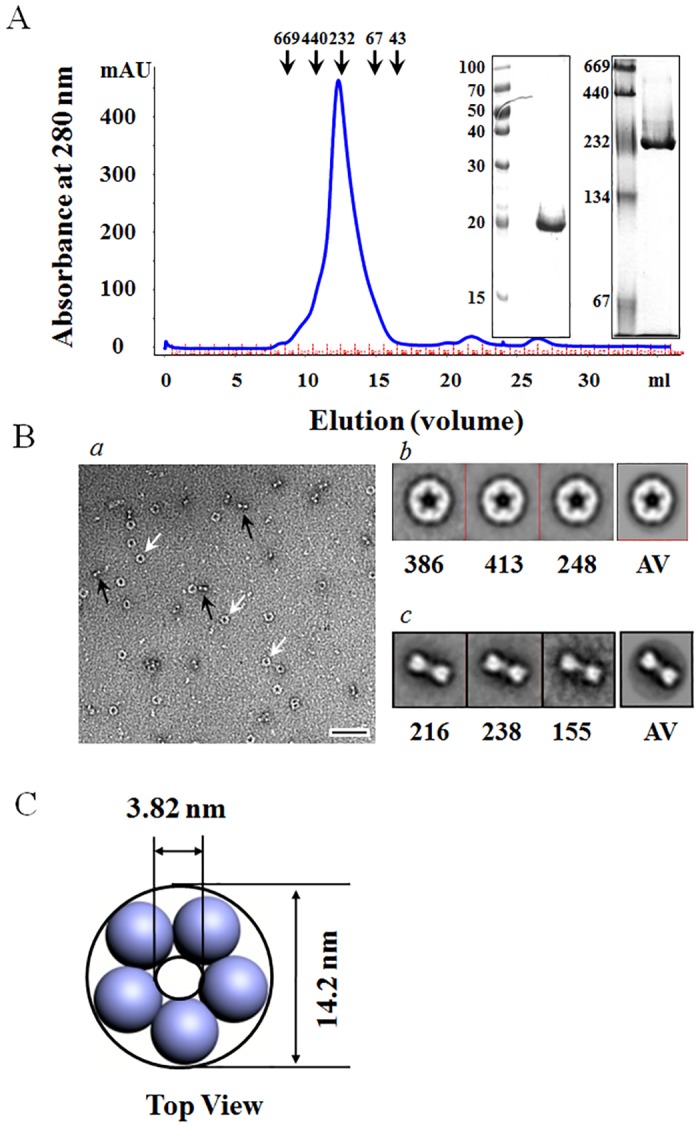
Structural Analysis of Recombinant 1-Cys TkPrx. (A) Purified 1-Cys TkPrx was analysed using a Superdex 200 10/300 GL column (left). The fractions were analysed by 8% BN-PAGE (right) and 12.5% SDS-PAGE (middle). (B) The appearance of 1-Cys TkPrx was visualized with electron microscopy followed by single particle analysis. (*a*) Negatively stained fields of 1-Cys TkPrx. Arrows indicate top (white) and side views (black) of its structure. (*b* and *c*) Global averages and selected class averages of decameric 1-Cys TkPrx were derived from the rotationally aligned images using the eleven most-significant eigenvectors, but without symmetrization. The numbers below the graphs indicate the particle amount used for image processing. AV indicates the correlation average of the top and side views. (C) Modelling of the top view based on image processing of 1-Cys TkPrx. The scale bar indicates 50 nm, and the magnification is 42,000x.

To determine the oligomeric state of 1-Cys TkPrx, we performed gel filtration chromatography, BN-PAGE and EM assays (Fig 2). Gel filtration analysis under non-denaturing conditions revealed that purified 1-Cys TkPrx has a molecular mass of ~230 (Fig 2A, left), which corresponds to a decameric form, assuming that the mass of each subunit is 24.6 kDa (Fig 2A, middle), as assigned by SDS-PAGE. The decamer is predicted to be the predominant form based on peak area analysis. Proteins from the main peak were further analysed by BN-PAGE followed by Coomassie staining, which produced results consistent with the gel filtration experiment (Fig 2A).

To further study the oligomeric states of 1-Cys TkPrx, we utilized TEM (Fig 2B). Electron micrographs of the negatively stained proteins from the peaks showed a uniform distribution, with the main peak consisting of a ring-shaped form and a dumbbell-shaped form (Fig 2B *a*). We subjected the protein particles from the main peak fraction to image processing to determine the folding states. A total of 1029 well-stained ring-shaped particles (Fig 2B *b*) and 609 dumbbell-shaped particles (Fig 2B *c*) were translationally and rotationally aligned and subjected to multivariate statistical analysis. For classification, we selected the three most-significant eigenvectors, resulting in the elimination of the less-significant information that was represented by the other eigenvectors. Using this approach, three classes were determined according to the similarity of their features. The class averages of the ring-shaped view revealed a structure with 5-fold symmetry (decamer) with heavy stain accumulation in its centre, while the dumbbell-shaped view revealed a 2-fold symmetry structure (Fig 2B). The diameter of the ring and the pore of the averaged decamer were approximately 14.2 and 3.82 nm, respectively. The width and height of the dumbbell-shaped structure were approximately 5.99 and 14.2 nm, respectively (Fig 2C). These results suggest that the observed dumbbell-shaped structure is a side-view of the ring-shaped 1-Cys TkPrx.

### Reduced and Oxidized forms of 1-Cys TkPrx

**Fig 3:**
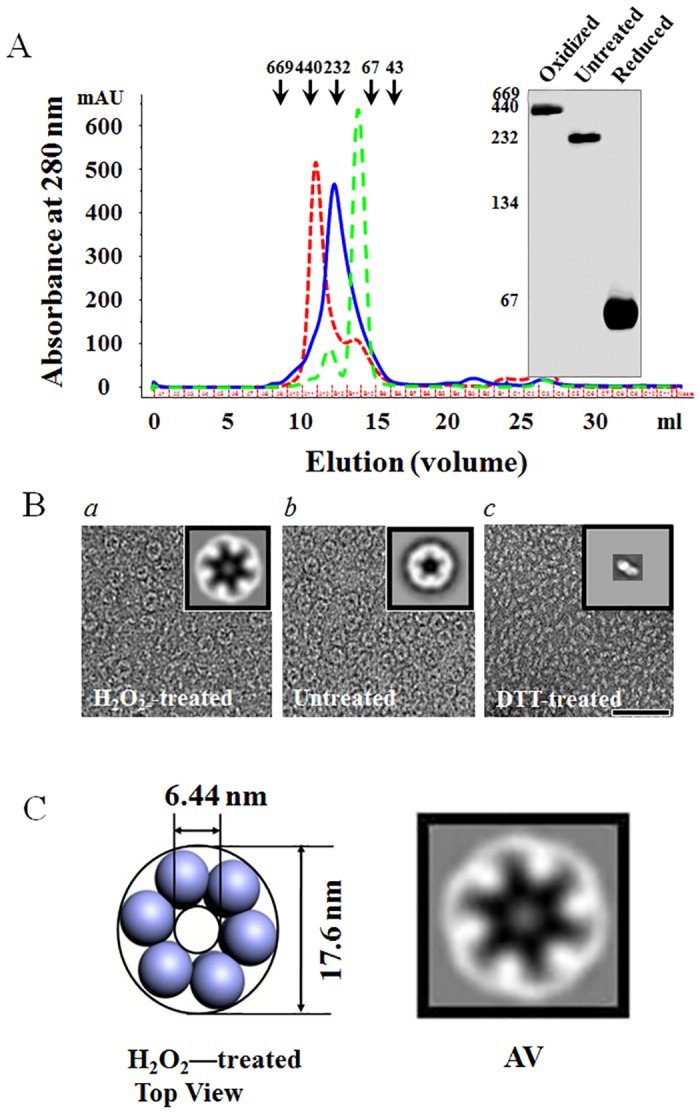
Reduced and Oxidized Forms of 1-Cys TkPrx. (A) Purified 1-Cys TkPrx was incubated with 1 mM DTT or 1 mM H_2_O_2_ for 12 h at 4°C and analysed using a Superdex 200 10/300 GL column (left) and 8% BN-PAGE (right). The variant lines show the different forms of 1-Cys TkPrx: 1 mM H_2_O_2_ (dotted line, red), untreated (solid line, blue), and 1 mM DTT (dotted line, green). (B) Negatively stained EM fields of 1-Cys TkPrx molecules (*a*) in the presence of 1 mM H_2_O_2_, (*b*) untreated (without any oxidizing and reducing agents), and (*c*) in the presence of 1 mM DTT. The insets show the correlation average of the top views. Averaged images of H_2_O_2_- or DTT-treated forms (B*a* and B*c*), produced from 780 and 951 images, respectively. The scale bar indicates 50 nm. (C) Modelling of the top view based on image processing of oxidized 1-Cys TkPrx. AV indicates the correlation average of the top view.

We examined whether 1-Cys TkPrx adopts different structures under different conditions because oxidative stress typically induces protein conformational changes in anaerobic archaea [27]. After incubating the samples in the presence of 1 mM H_2_O_2_ or DTT at 85°C, H_2_O_2_ or DTT was removed by ultrafiltration. The proteins were analysed by gel filtration, BN-PAGE and electron microscopy. Gel filtration showed that the molecular masses of untreated 1-Cys TkPrx, oxidized 1-Cys TkPrx and reduced 1-Cys TkPrx were approximately 240, 300, and 70 kDa, respectively (Fig 3A). Furthermore, BN-PAGE analysis was consistent with the gel filtration results.

We then performed EM to further examine the oligomeric states of 1-Cys TkPrx (Fig 3B). The electron micrographs and image processing showed that oxidized 1-Cys TkPrx formed a dodecamer. The diameter of the protein and the size of the pore both increased, to approximately 17.6 and 6.44 nm, respectively (Fig 3C). However, the DTT-treated 1-Cys TkPrx did not exhibit a uniform structure in the EM graphs, and the protein was predicted to be a dimer based on BN-PAGE and gel filtration analysis (Fig 3A and 3B).

### Point mutations induce conformational changes in 1-Cys TkPrx

**Fig 4:**
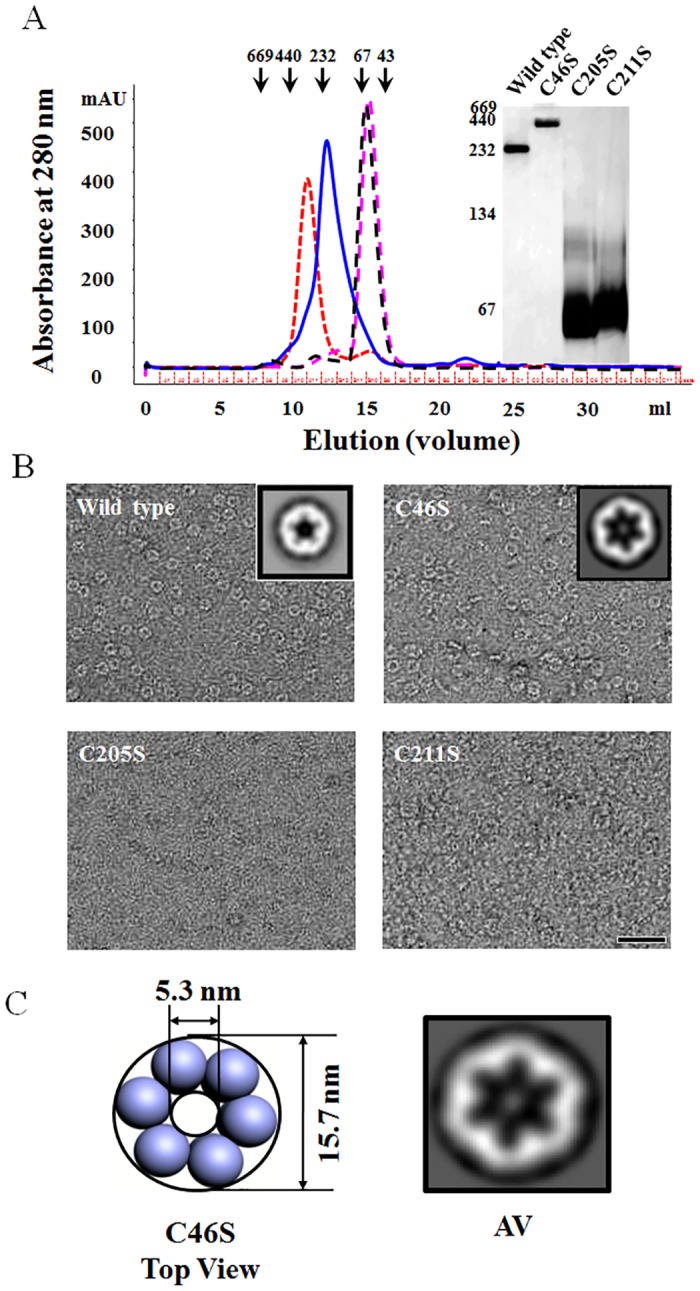
Structural analysis of 1-Cys TkPrx mutants. (A) Wild type and mutants were analysed using a Superdex 200 10/300 GL column (left) and 8% BN-PAGE (right). The variant lines indicate the different mutants of 1-Cys TkPrx: Wild type (solid line, blue), mutant C46S (dotted line, red), mutant C205S (long dashed line, magenta) and mutant C211S (long dashed line, black). (B) Appearance of the negatively stained wild type and mutant proteins. Average images of C46S, C205S, and C211S produced from 1205, 122, and 176 images, respectively. The scale bar indicates 50 nm, and the magnification is ×42,000. (C) Modelling of the top view based on image processing of the C46S mutant protein of 1-Cys TkPrx.

1-Cys TkPrx likely adopts different oligomeric structures depending on the reduction and oxidation conditions. Because cysteines are very sensitive to oxidative stress, we analysed how the cysteine residues contribute to the different oligomeric states. To test this, we mutated the three cysteines (C46, C205, and C211) to serines. We found that the C46S mutant protein forms a dodecamer structure, as determined by gel filtration, BN-PAGE and TEM analysis (Fig 4A and 4B). EM and image processing results showed that the diameter and pore size of the C46S mutant protein were 15.7 and 5.3 nm, respectively (Fig 4C), similar to the oxidized form of 1-Cys TkPrx (Fig 3B and 3C). Meanwhile, the molecular masses of the other mutants (C205S and C211S) were determined to be ~60 kDa by gel filtration and BN-PAGE (Fig 4A). Neither of the two mutants formed regular structures or high oligomeric states in the EM graphs. The cysteines in these two mutants were located in the C-terminal CXDWWFC motif that is highly conserved in archaeal Prxs (S1 Fig). However, the function of this conserved motif of archaeal Prxs has not been previously reported. Our data suggest that the CXDWWFC motif of a thermophilic Prx may influence high-molecular-weight oligomer formation.

### The dual functions of 1-Cys TkPrx are regulated by changes in protein structure

**Fig 5:**
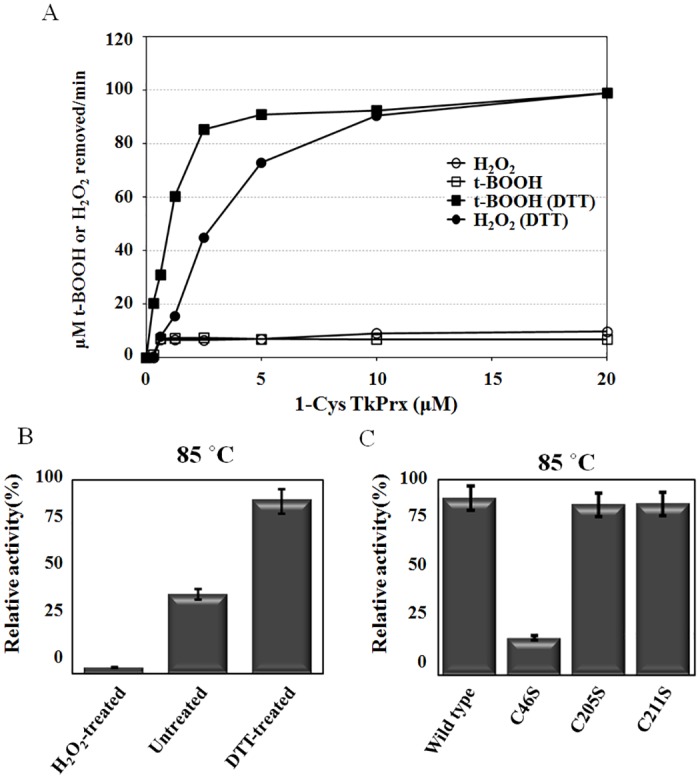
Peroxidase Activity of the Various States of 1-Cys TkPrx. (A) 1-Cys TkPrx metabolizes H_2_O_2_ and t-BOOH in a protein-concentration-dependent manner. The protein was incubated with the substrates H_2_O_2_ (circles) and t-BOOH (squares) in the presence (closed) or absence (open) of DTT at 85°C. Each point represents the mean of three separate experiments. (B) The peroxidase activity of the oxidized form (H_2_O_2_-treated), the untreated form, and the reduced form (DTT-treated) of 1-Cys TkPrx at 85°C. (C) Comparison of the peroxidase activity of wild type, the C46S mutant, the C205S mutant, and the C211S mutant at 85°C.

To investigate the physiological function of the cysteines in the catalysis of 1-Cys TkPrx, we measured the peroxidase activity of the wild type protein and various mutants with a FOX assay (Fig 5). Organic (t-BOOH) or inorganic (H_2_O_2_) hydroperoxides were incubated with 1-Cys TkPrx in an enzyme-concentration-dependent manner. Fig 5A shows that 1-Cys TkPrx can act as an organic and inorganic hydroperoxide peroxidase. In addition, the dimeric 1-Cys TkPrx (DTT-treated) exhibited stronger decomposable activity of H_2_O_2_ than the decameric (untreated) or dodecameric (H_2_O_2_-treated) forms at the physiological temperature of *T*. *kodakarensis* KOD1 (Fig 5B). Comparisons between the wild type enzyme and the mutants showed that the C205S and the C211S mutant proteins have Prx activity similar to that of the wild type protein; however, the C46S mutant had < 10% of the peroxidase activity of the wild type protein (Fig 5C). These results further suggest that the cysteine in the conserved C-terminal CXDWWFC motif is not responsible for catalysis.

**Fig 6:**
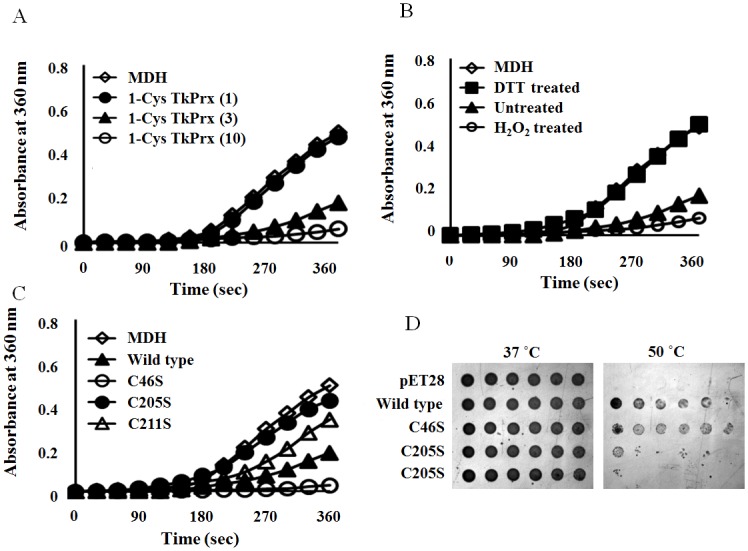
Chaperone Activity of 1-Cys TkPrx. (A) The chaperone activity of wild type 1-Cys TkPrx. MDH (open diamonds, 1.7 μM) was incubated with 1.7 μM 1-Cys TkPrx (closed circles), 5.4 μM 1-Cys TkPrx (closed triangles) and 17 μM 1-Cys TkPrx (open circles) at 85°C. (B) The chaperone activity of the oxidized form (open circles, 5.4 μM), the untreated form (closed triangles, 5.4 μM), and the reduced form (closed circles, 5.4 μM) of 1-Cys TkPrx at 85°C. MDH without 1-Cys TkPrx (open diamonds, 1.7 μM) was used as a negative control. (C) Comparison of the chaperone activity of wild type (closed triangles, 5.4 μM), C46S mutant (open circles, 5.4 μM), C205S mutant (closed circles, 5.4 μM) and C211S mutant (open triangles) at 85°C. MDH alone (open diamonds, 1.7 μM) was used as a control. The data shown represent the mean of at least three independent experiments. (D) 1-Cys TkPrx protects the *E*. *coli* cells from heat shock. *E*.*coli* cells expressing either wild type or mutant 1-Cys TkPrx were grown at 37°C for 24 h or preheated at 50°C for 30 min and then incubated at 37°C for 24 h. Various dilutions (10^–1^ to 10^–6^) were spotted on LB plates containing 1 mM IPTG. *E*.*coli* cells containing the pET28 vector were used as a control.

We considered that the non-catalytic form of 1-Cys TkPrx might play another role, similar to the dual functions of the 2-Cys Prxs as both peroxidases and molecular chaperones [7]. To verify whether 1-Cys TkPrx has both peroxidase and molecular chaperone activity, we next assayed the chaperone activity of 1-Cys TkPrx using *T*. *flavonus* MDH as a substrate. 1-Cys TkPrx prevented the thermal aggregation of MDH at 95°C in a dose-dependent manner, and aggregation was completely blocked at a subunit molar ratio of 1:10 (MDH:1-Cys TkPrx) (Fig 6A). The dodecameric complex (H_2_O_2_-treated) exhibited a high level of chaperone activity, while the DTT-treated enzyme did not exhibit chaperone activity (Fig 6B). This indicates that the molecular chaperone activity of 1-Cys TkPrx was significantly affected by the change in protein structure. We also measured the chaperone activity of the different mutants to confirm the relationship between 1-Cys TkPrx structure and function. The results showed that the dodecameric C46S form exhibited a much higher level of chaperone activity than the low-molecular-weight mutants C205S and C211S (Fig 6C). We also performed an *E*. *coli* dilution bioassay on LB agar using the cells expressing wild type 1-Cys TkPrx and the mutants. The *E*. *coli* expressing wild type and the C46S mutant 1-Cys TkPrx displayed higher tolerance to heat stress (Fig 6D). Moreover, these results were in excellent agreement with the *in vitro* chaperone activity assay. Therefore, the dual functions of 1-Cys TkPrx are closely related to structural changes. We propose that the low-molecular-weight 1-Cys TkPrx metabolizes organic or inorganic hydroperoxide and reduces the efficiency of peroxidase, whereas the dodecameric 1-Cys TkPrx promotes molecular chaperone activity.

### 1-Cys TkPrx can bind to DNA

**Fig 7:**
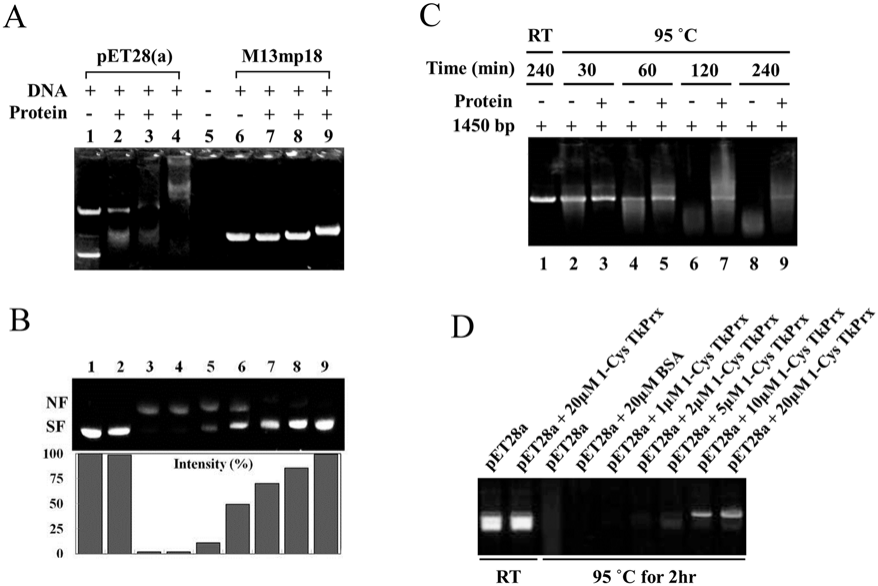
1-Cys TkPrx Prevents Oxidative and Thermal DNA Damage. (A) The ability of 1-Cys TkPrx to bind DNA. Double-stranded (5369 bp, 100 ng) or single-stranded (7249 bp, 100 ng) plasmid DNA was incubated with 1-Cys TkPrx in 25 mM Tris-HCl (pH 7.4) buffer containing 50 mM NaCl, 0.5 mM EDTA, 1 mM MgCl_2_, and 0.5 mM DTT at room temperature for 30 min, and then aliquots were taken for analysis on a 1% agarose gel. pET28(a) as double-stranded plasmid DNA was mixed with low-molecular-weight (lane 2), decameric (lane 3), and dodecameric (lane 4) forms of 1-Cys TkPrx. pET28(a) alone (lane 1) was used as a control. M13mp18 as single-stranded plasmid DNA was mixed with low-molecular-weight (lane 7), decameric (lane 8), and dodecameric (lane 9) forms of 1-Cys TkPrx. M13mp18 alone (lane 6) was used as a control. (B) The effects of 1-Cys TkPrx in protecting the supercoiled structure of pET plasmid DNA against oxidative damage in the MCO system. Lane 1, plasmid DNA alone; lane 2, plasmid DNA and Fe^3+^; lane 3, plasmid DNA and the MCO system; lane 4, plasmid DNA, 40 μM BSA, and the MCO system; lane 5–9, plasmid DNA, the MCO system and differing amounts of 1-Cys TkPrx (1, 5, 10, 20, and 40 μM, respectively). The bands corresponding to the nicked form (NF) and the supercoiled form (SF) of the plasmid DNA are indicated on the right-hand side. The lower panel shows the density of SF. (C and D) 1-Cys TkPrx can protect DNA from thermal stress. (C) Linear dsDNA (1945 bp) and 1-Cys TkPrx were mixed and then heated (thermal stress) at 95°C for 30, 60, 120, and 240 min to hydrolyse the DNA. dsDNA at room temperature was used as a control. (D) pET28(a) plasmid DNA was mixed with different concentrations of 1-Cys TkPrx and incubated at 95°C for 2 h. BSA (20 μM) was used as a negative control. The aliquots were visualized on a 1% agarose gel.

Previous experimental evidence suggested that bacterioferritin comigratory protein, which belongs to the peroxidase family of proteins in *Coxiella burnetii*, was a potential DNA-binding protein [28]. We therefore investigated whether 1-Cys TkPrx interacts with DNA using an electrophoretic mobility shift assay (EMSA) (Fig 7A). Oxidized, untreated and reduced wild type 1-Cys TkPrx were incubated with double-stranded (pET28(a)) and single-stranded (M13mp18) plasmid DNA for 1 h, and the protein-DNA mixtures were analysed by 1% agarose gel. The results revealed that oxidized TkAhpC could bind DNA tightly, while the reduced 1-Cys TkPrx bound DNA weakly. Furthermore, the mobility of the double-stranded plasmid DNA was much lower than the mobility of the single-stranded plasmid DNA. To investigate whether DNA length affects the binding ability of 1-Cys TkPrx, we synthesized a series (10–80 bp, 100–1500 bp) of polynucleotides and incubated them with 1-Cys TkPrx for EMSA. The retardation of DNA bands was observed for nucleotides greater than 300 bp, and low-molecular-weight DNA-protein binding was not observed (data not shown). These results suggest that the dodecameric form of 1-Cys TkPrx preferably binds to double-stranded plasmid DNA.

### 1-Cys TkPrx protects DNA from hydroxyl radicals and thermal damage

An MCO assay was used to investigate whether 1-Cys TkPrx can protect other macromolecules from hydroxyl radicals. The level of DNA nicking by hydroxyl radicals was determined by measuring the mobility of the DNA molecules on an agarose gel (Fig 7B). The results showed that hydroxyl radicals from the MCO system could induce damage in supercoiled DNA. In the absence of 1-Cys TkPrx, 97% of the supercoiled plasmid DNA was nicked. When plasmid DNA was mixed and incubated with various concentrations of 1-Cys TkPrx (1–40 μM), we found that 1-Cys TkPrx could protect the supercoiled form of the plasmid DNA. These results demonstrate that 1-Cys TkPrx exhibits antioxidant activity by protecting DNA from hydroxyl radicals.

To investigate if 1-Cys TkPrx can protect DNA under other stress conditions, we incubated 1-Cys TkPrx with a 1945-bp linear dsDNA and pET28(a) plasmid DNA for different amounts of time at 95°C (Fig 7C and 7D). With increasing incubation periods, linear dsDNA was gradually degraded in the absence of 1-Cys TkPrx. However, 1-Cys TkPrx could protect the DNA from thermal damage, even when the DNA was exposed to 95°C for 240 min (Fig 7C). Like the linear dsDNA, pET28(a) plasmid DNA was also degraded in the absence of 1-Cys TkPrx. 1-Cys TkPrx (10 μM) could protect the plasmid DNA over a 2 h incubation at 95°C, but 20 μM of BSA (as a negative control) could not protect the plasmid DNA. These results indicate that 1-Cys TkPrx can protect DNA from thermal stress.

## Discussion

Here, we identified *T*. *kodakaraensis* KOD1 proteins that were up-regulated by oxidative, osmotic, and heat stress through 2DE analysis and MALDI-TOF MS. Prxs are thiol-based peroxidases that are widely distributed in all living organisms, from bacteria to mammals [9], and can detoxify H_2_O_2_ through reduction to water and alcohols [29]. 1-Cys TkPrx was identified as a peroxidase belonging to the 1-Cys Prx family of proteins and can decompose H_2_O_2_ and t-BOOH (Fig 5A and 5B). To elucidate the reaction mechanism of this protein in more detail, sequence alignment, mutation analysis, and structure-dependent peroxidase activity analysis were performed. The results showed that C46S lost peroxidase activity and obtained chaperone activity compared to the wild type protein and the C205S and C211S mutants. This phenomenon is similar to the function of 2-Cys Prx in plants. The C54S mutant of 2-Cys Prx in plants predominantly forms decamers and has chaperone activity [30]. Analysis of its amino acid sequence revealed that 1-Cys TkPrx has three cysteine residues in the N-terminus and C-terminus. The peroxidatic cysteine (Cys^46^) located in the N-terminus scavenges hydroperoxides, and the oxidizing cysteine induces the formation of higher oligomeric structures such as decamers and dodecamers, while the two cysteine residues (Cys^205^ and Cys^211^) located in the C-terminus are important for oligomerization because the mutant proteins that contained cysteine-to-serine substitutions at the C-terminus formed dimers.

We examined the structural and functional properties of untreated 1-Cys TkPrx and found that 1-Cys TkPrx is the decameric form. A gene (APE2278) encoding a Prx was identified in the genome database of the aerobic hyperthermophilic archaeon *A*. *pernix*, which encodes a hexadecameric protein composed of two identical octamers. However, its chaperone function has not been identified [16]. A previous study suggested that Prxs might act as a molecular switch, such that their peroxidase and molecular chaperone functions are dependent on structural changes [7, 31]. The structural change of *Schistosoma mansoni* 2-Cys Prx induced by H_2_O_2_ is related to (1) overoxidation of the sulfur atom of Cys, (2) concerted tertiary variations, and (3) quaternary structural changes [31]. Similarly, 1-Cys TkPrx also appears to depend on structural changes induced by oxidation and reduction. One millimolar H_2_O_2_, which is enough to oxidize Cys residues to their sulfinylated and sulfonylated forms in Prxs [32, 33], can induce 1-Cys TkPrx to take its dodecamer forms (Fig 3). If 1-Cys TkPrx was treated by 10 mM H_2_O_2_, the oxidized protein also displayed dodecameric forms and had chaperone activity similar to 1-mM-H_2_O_2_-treated proteins (data not shown). Conclusively, the decamer, or higher order forms such as the dodecamer of 1-Cys TkPrx, functions as a molecular chaperone that can protect against protein aggregation induced by thermal stress, while the dimer has peroxidase activity. The chaperone function of the C46S variant suggests that the chaperone activity does not necessarily depend on the oxidized Cys in the protein. Hyperoxidation represents only one possibility for switching the function from peroxidase to chaperone. The peroxidase and chaperone functions of 1-Cys TkPrx are consistent with our previous research [7, 34], which strongly suggests that the mechanism of Prx function is conserved from the archaea to eukaryotic cells.

We also showed that 1-Cys TkPrx could protect DNA from damage caused by hydroxyl radicals and high temperature. This protection may be due to two reasons. First, 1-Cys TkPrx can protect DNA by its peroxidase activity. A previous study has reported that human and silkworm Prxs can remove hydroxyl radicals produced by the MCO system using DTT as a thiol source [35, 36]. In our study, 1-Cys TkPrx could maintain the supercoiled form of plasmid DNA in the MCO system, which may be because the hydroxyl radicals were reduced by 1-Cys TkPrx (Fig 7B). Second, 1-Cys TkPrx exhibits DNA-binding activity, and the oxidized dodecameric 1-Cys TkPrx can bind DNA more tightly than other forms. The protein-DNA complex may also protect DNA from damage (Fig 7). These results clearly indicate that 1-Cys TkPrx is a moonlighting enzyme that can perform more than one function and that these dual functions do not depend on gene fusions, splice variants, or post-translational modifications; moonlighting proteins also exclude families of homologous proteins, proteins capable of utilizing multiple substrates, and proteins catalysing multiple steps in the same metabolic pathway [7, 34]. The moonlighting function of 1-Cys TkPrx is conserved from archaea to eukaryotes, which is similar to the glyceraldehyde-3-phosphate dehydrogenase in *T*. *kodakarensis* KOD1 that may cause cell death if it functions as in eukaryotes [37]. This also suggests that moonlighting enzymes may be conserved during evolution and that moonlighting in archaea may represent a primeval step in evolution.

## Supporting Information

S1 Fig Primary Sequence Alignment of 1-Cys TkPrx with Homologous Prxs. (DOCX)
