# Supplementary material for: A 1-Cys Peroxiredoxin from a Thermophilic Archaeon Moonlights as a Molecular Chaperone to Protect Protein and DNA against Stress-Induced Damage
Source: PLoS One. 2015 May 1;10(5):e0125325. doi: 10.1371/journal.pone.0125325 (PMC4416765; doi:10.1371/journal.pone.0125325)
Supplement: S1 Fig — (DOCX) [file pone.0125325.s001.docx]

**Supplemetary data**


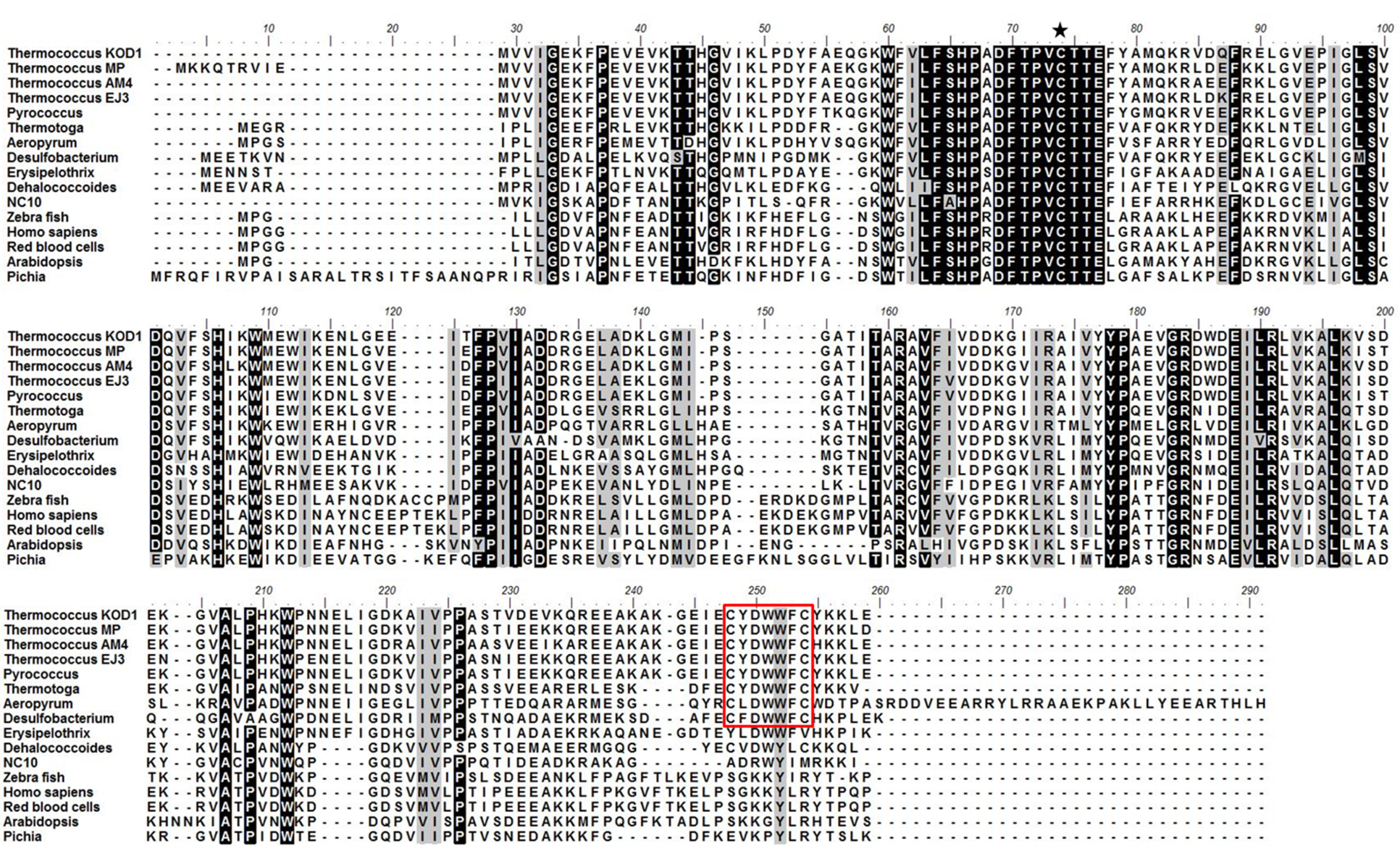


**S1 Fig. Primary Sequence Alignment of 1-Cys TkPrx With Homologous Prxs.** The sequence of archaeal Prxs(1-Cys) from *Thermococcus kodakarensis* KOD1(1-Cys TkPrx, YP_182950.1), *Thermococcus barophilus* MP(YP_004070545.1), *Thermococcus sp.* AM4(ZP_04880389.1), *Thermococcus gammatolerans* EJ3(YP_002959619.1), *Pyrococcus horikoshii* OT3(NP_143112.1), *Thermotoga petrophila* RKU-1(YP_001243726.1), and *Aeropyrum pernix* K1(Q9Y9L0.1); prokaryotic Prxs(1-Cys) from *Desulfobacterium autotrophicum* HRM2(YP_002605131), *Erysipelothrix rhusiopathiae str.* Fujisawa(YP_004560275), *Dehalococcoides sp.* CBDB1(YP_308595), and *NC10 bacterium* ‘Dutch sediment’(CBE69035); eukaryotic Prxs(1-Cys) from *Danio rerio* (Zebra fish, AAH59671), *Homo sapiens* (AAH53550), *Red blood cells page spot* 12 (P30041), *Arabidopsis thaliana* (AEE32252), and *Pichia pastoris* GS115 (CAY70965) were aligned by the program Clustal W. The “CYDWWFC” motif was highly conserved in the archaeal Prxs. The motif was marked with a box. The asterisk above the primary sequence indicates complete conservation with the other Prxs.
